# Effect of chemotherapy on ovarian function in pre-menopausal women with breast cancer

**DOI:** 10.3892/mi.2025.279

**Published:** 2025-10-27

**Authors:** Olaiya Popoola, Mohammad Habeebu, Akeem Adeniji, Olukemi Alegi, Gbenro Olukiran, Uchenna Okoro, Oladimeji Adepoju, Atara Ntekim

**Affiliations:** 1 Department of Radiation and Clinical Oncology, Marcelle Ruth Cancer Center, Lagos 101241, Nigeria; 2 Department of Radiotherapy (Oncology) Lagos University Teaching Hospital, Lagos 112005, Nigeria; 3 Department of Radiation and Clinical Oncology, MeCure Cancer Center, Lagos 100261, Nigeria; 4 Department of Radiation and Clinical Oncology, Federal Medical Center, Lagos 101212, Nigeria; 5 Department of Medical Services, Lagos State Primary Health Care Board, Lagos 100001, Nigeria; 6 Department of Radiation Oncology, College of Medicine, University of Ibadan, Ibadan 200221, Nigeria

**Keywords:** breast cancer, chemotherapy, pre-menopausal, hormones, fertility, ovarian function

## Abstract

Chemotherapy has been known to negatively affect ovarian function. This sometimes results in infertility, particularly in young patients who still desire to have children. The effect of chemotherapy on young women with breast cancer has not been extensively studied. The objective of the present study was to determine the effects of chemotherapy on ovarian function/reserve among pre-menopausal female patients with breast cancer. Pre-menopausal females with breast cancer scheduled for chemotherapy were recruited for the study. Blood samples were obtained to determine the plasma concentrations of three hormones, namely estrogen, follicle stimulating hormone and anti-Müllerian hormone. The samples were collected at pre-chemotherapy, at 3 months and then at 6 months post-chemotherapy. The assays were performed using the direct immunoenzymatic colorimetric method. Data from 82 participants were analyzed. The age range of the patients was 32-41 years (mean age, 36.6±4.6 years). There were significant reductions in the plasma concentrations of estrogen and anti-Müllerian hormone, with significant increase in the levels of follicle stimulating hormone. An irregular or the cessation of menstrual flow was observed in some patients who were >30 years of age. There was no significant difference in the effects with different chemotherapeutic regimens for breast cancer. The negative ovarian effects on tested hormones persisted 6 months post-chemotherapy. Overall, the present study demonstrates that chemotherapy for breast cancer has deleterious effects on ovarian function, as reflected by altered plasma levels of estrogen, follicle stimulating hormone and anti-Müllerian hormone. Clinicians should be aware of this and counsel such patients appropriately on fertility issues to ensure a better quality of life for patients who survive breast cancer.

## Introduction

Breast cancer is the most common malignancy in Nigeria. The incidence of breast cancer increases with age, with the majority of women diagnosed >40 years of age (1). Globally, ~25% of women with breast cancer are pre-menopausal and peri-menopausal, while the remaining are post-menopausal. A great burden of pre-menopausal women with breast cancer are found in low-income and middle-income countries with 55.2% of total breast cancer cases in countries with a low human development index occurring in pre-menopausal women (2). In Nigeria, the proportion of breast cancer among pre-menopausal women is close to 60% (3). This group therefore has a higher proportion of breast cancer cases among individuals of African ancestry, more than the proportion among Caucasians, where young women of reproductive age account for the minority of women that are diagnosed with breast cancer. There is therefore a racial difference in terms of the incidence of young women with breast cancer. It has been reported that prior to the age of 35 years, the percentage of African Americans with breast cancer is more than twice that of the Caucasians (4).

Although the incidence of breast cancer has progressively increased recently, there has also been a simultaneous improvement in the survival rates. This is partly due to early detection and adjuvant chemotherapy (5). Despite improved survival rates however, there are certain issues associated with survivorship, such as fertility concerns, late side-effects of treatment, as well as psychosocial issues. Pre-menopausal women are those with a regular menstrual period. Pre-menopausal women pass through different levels of monthly cyclic changes, which are characterized by varying hormonal levels that signal each phase of the menstrual cycle.

Hormonal levels in pre-menopausal women for estrogen and progesterone, luteinizing hormone (LH), anti-Müllerian hormone (AMH) and follicle stimulating hormone (FSH) fluctuate during the menstrual cycle, following the pattern presented in Table I (6).

The peri-menopausal state is regarded as the time during which a women has irregular menstruation cycles; menstruation can cease for ≥3 months and then resumes. A woman is said to be menopausal after having attained the complete cessation of menstrual periods for at least 12 months (7,8).

Young women with breast cancer are considered to be high-risk subjects as they are likely to have estrogen receptor-negative/high-grade tumors; therefore, chemotherapy is commonly part of their treatment modalities. The majority of chemotherapeutic regimens for breast cancer have deleterious effects on the ovaries, thereby affecting fertility. These women may also attain a state of premature amenorrhea, which can be temporary or permanent. Women who resume regular menstrual cycles following the completion of chemotherapy may be less fertile compared with those who have not received any chemotherapy. They may also attain menopause earlier than expected (premature menopause) (9-11). The rate of amenorrhea varies from 21 to 71% in young women and 49-100% in those >40 years of age treated with chemotherapy (12).

Fertility concerns are major considerations in women of childbearing age who are placed on chemotherapy, particularly among those who still desire to have children. On this account, concerns for fertility may render treatment decision-making more complex for young women with breast cancer (13), particularly as young women with breast cancer experience greater psychosocial distress and anxiety about infertility compared with their older counterparts.

Primary ovarian insufficiency and premature menopause are regarded as potential compromises on reproductive potential. Sometimes a woman may menstruate, but may still suffer from partial ovarian injury, which may result in subfertility (14). Due to the increasing incidence of breast cancer among pre-menopausal women, some patients are concerned about how they will successfully conceive following the completion of treatment, which includes the administration of chemotherapy. Clinicians, reproductive medicine specialists and gynecologists should be conversant with these concerns of the patients.

Research has demonstrated that the concentrations of female sex hormones, such as AMH, inhibin-B, inhibin-A, FSH, LH and estradiol (E_2_) exhibit various changes following the administration of chemotherapy (15-18). To date, reports on the effects of chemotherapy on female sex hormones (which are a reflection of ovarian function) among indigenous populations of African origin are limited, hence the need for the present study. The aim of the present study was to determine the effects of chemotherapy on ovarian function/reserve among the study population which included a high proportion of pre-menopausal women, most of whom desired child birth following chemotherapy. The levels of three hormones, namely FSH, E_2_ and AMH were measured. AMH is relative stable, without it being influenced by the menstrual cycle; hence researchers have selected AMH as a reliable biomarker to assess ovarian reserve during and after chemotherapy (19) (Table I).

## Patients and methods

The present study was a prospective study conducted at the Department of Radiation and Clinical Oncology of the Lagos University Teaching Hospital, Idi-araba, Lagos, Nigeria, between October, 2020 to November, 2023. Young women within the reproductive age group (18-42 years) and with a histologically confirmed diagnosis of breast cancer were counseled appropriately on the study and their written consent for participation in the study was obtained. Participants were counselled on fertility preservation and asked for their preferences.

Blood samples were collected from eligible pre-menopausal women after obtaining consent, that were newly diagnosed with breast cancer and who were planning to receive chemotherapy as part of their oncological treatment. The samples were collected prior to treatment with chemotherapy (3-5 days from the last menstrual period), and then at 3 and 6 months following the completion of chemotherapy. Patients were also followed-up for 12 months to observe menstrual flow patterns. The patients used a menstrual diary to track their date and duration of flow. However, the flow quantity was not measured. The number of sanitary pads used during each menstrual period was recorded and used to estimate the quantity of menstrual flow, while the duration of flow was also recorded. All the patients recruited for the study were on a 3-week chemotherapeutic regimen. First-line chemotherapy was administered every 3 weeks amounting to four courses within the first 12 weeks. This consisted of an anthracycline (doxorubicin 60 m^2^ or epirubicin 75 mg/m^2^) + cyclophosphamide at 600 mg/m^2^ with or without a 5-fluorouracil regimen at 600 mg/m^2^ (AC/CAF) or docetaxel at 75 m/m^2^ + doxorubicin at 60 mg/m^2^ + cyclophosphamide at 600 mg/m^2^ (TAC) regimen. In the second 12 weeks, participants received four courses administered every 3 weeks of either a taxane (docetaxel at 75 mg/m^2^ or paclitaxel at 175 mg/m^2^) + capecitabine at 2,000 mg twice daily or a platinum compound (cisplatin at 50 mg/m^2^ or carboplatin 5 AUC) + a taxane regimen (PT) as a second regimen based on institutional guidelines. The blood samples obtained from the participants were stored and analyzed at the Human Reproductive and Endocrinology Research Laboratory, Department Obstetrics and Gynecology, College of Medicine, University of Lagos/Lagos University Teaching Hospital (LUTH), Idi-Araba, Lagos, Nigeria. Ethical approval for the study was obtained from institutional Ethics Committee at Lagos University Teaching Hospital Health Research Ethics Committee with the assigned no. ADM/DCST/HREC/APP/2736. Written and signed informed consent was obtained from the participants prior to enrolment in the study.

The participants included in the study were females, aged 18-42 years, who had histologically confirmed breast cancer at stages 1-3, who were chemotherapy naïve and with an ECOG performance status of 0-1. The exclusion criteria were pregnant patients, those who had been previously treated with chemotherapy, those who had undergone a hysterectomy, as well as those with an irregular menstrual cycle, as well as post-menopausal women or with last menses >3 months prior.

### Sample collection and laboratory analyses

The specimens were blood samples collected by venipuncture into lithium heparin bottles. Of note, ~5 ml blood samples were obtained from each participant with minimal pain at the site of venipuncture. The blood samples collected were centrifuged at 2,000 x g for 10 min at 4˚C to obtain plasma; these samples were stored in a freezer at -80˚C and later analyzed.

In total, three different sets of blood samples were collected for the study: Prior to the commencement of chemotherapy, at 3 months following the completion of chemotherapy and at 6 months following chemotherapy. The chemotherapy was administered at 3 weekly intervals. The plasma samples were stored at -80˚C and analyzed in the laboratory for AMH, E_2_ and FSH. A direct immunoenzymatic colorimetric method was used for the quantitative determination of AMH, E_2_ and FSH concentrations in plasma using ELISA kits (cat. nos. SEK-0003, SEK-0008 and SEK-0010, Rapid Labs) as previously described (15).

### Statistical analysis

Data analysis was carried out using SPSS version 23 software (IBM Corp.). The descriptive characteristics of the study participants were summarized using tables and charts. Numerical data, including the levels of hormonal parameters among the study participants are expressed as the mean (SD) at pre-chemotherapy, at 3 months post-chemotherapy and at 6 months post-chemotherapy. The effects of the various chemotherapy combinations on hormone levels were analyzed using paired t-tests at two specific intervals: Pre-chemotherapy vs. 3 months post-chemotherapy, and 3 vs. 6 months post-chemotherapy. Repeated measures analysis of variance (ANOVA) was used to compare the hormone levels (AMH, E_2_ and FSH) measured in the same study participants at three different time intervals [pre-chemotherapy (baseline), at 3 months post-chemotherapy and at 6 months post-chemotherapy]. Where significant differences were observed, pairwise comparisons between time points were conducted using Bonferroni post hoc tests to control for multiple comparisons. A Fisher's exact test was conducted to assess the association between age group and menstruation patterns at 6 months following chemotherapy. A value of P<0.05 was considered to indicate a statistically significant difference.

## Results

A total of 96 pre-menopausal women diagnosed with breast cancer were recruited for the present study. However, 14 dropped out of the study and were exempted from the analysis. The reasons for dropping out included seeking alternative treatments, such as spiritual and herbal healing, pregnancy, financial constraints and a change of residence. Parameters from 82 participants were analyzed. The mean age of all the participants in the study was 36.6 years (range, 32-41 years). The majority (71%) had at least 1 child, while 29% were nullipara. Of note, ~51% of the patients still desired to have more children. The age of the participants ranged from 32-41 years, with a mean age at diagnosis of breast cancer of 35.5 years (Table II). As regards the preference for fertility preservation, 63 (77%) were willing to use methods to preserve fertility, while 19 (23%) declined. Preferred fertility methods were as follows: The use of drugs 38 (46%); ovum banking 13 (16%) and adoption 25 (31%), while 6 (7%) patients did not choose any specific method of fertility preservation. Unfortunately, none of those who preferred fertility preservation could afford goserelin, which is the least costly form of fertility preservation currently available in Nigeria.

Most of the participants had AJCC anatomic stage III disease. Other clinical parameters of participants are presented in Table III. The first line of chemotherapy administered within the first 12 weeks consisted of anthracycline + cyclophosphamide/5-fluorouracil regimen (AC/CAF) (81 participants), or the taxane + anthracycline + cyclophosphamide (TAC) regimen (only 1 participant). In the second 12 weeks, 61 participants continued with AC/CAF, no participant continued with the TAC regimen, while 13 participants had their chemotherapy changed to taxane + xeloda, and 8 patients received the platinum + taxane regimen (PT) based on response and toxicity evaluations following institutional guidelines (Table IV). The plasma levels of the hormones of interest, namely AMH, E_2_ and FSH exhibited variations during the study period, as demonstrated in Table V.

**Figure 1:**
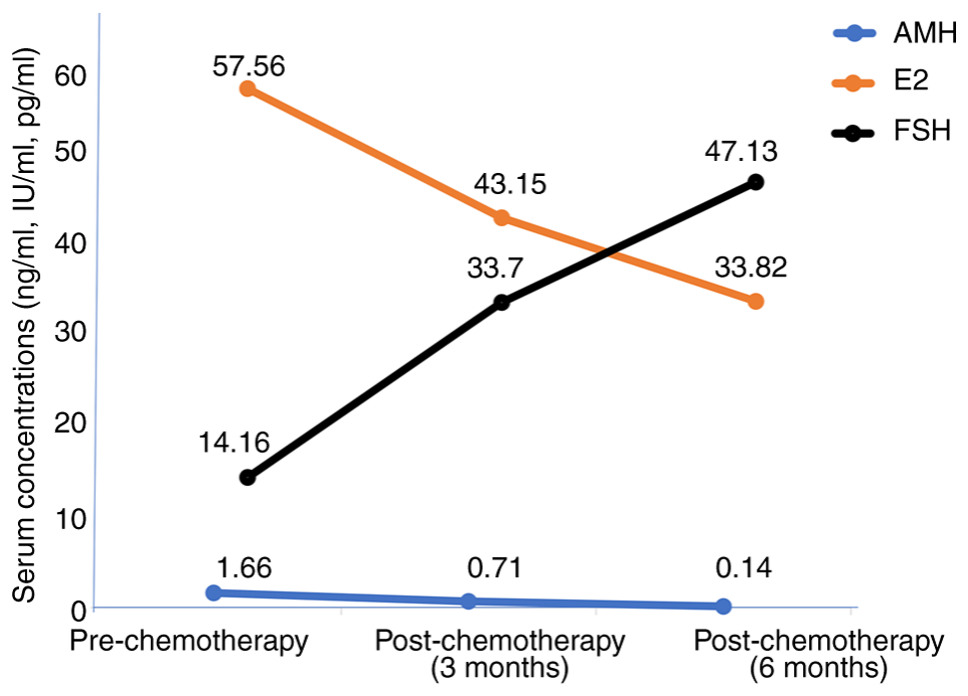
Trends in the levels of the hormones during the study period. AMH (P<0.001), E_2_ (P=0.003) and FSH (P<0.001). AMH, anti-Müllerian hormone; LH, luteinizing hormone; FSH, follicle stimulating hormone.

The trend of the levels of the hormones during the study period is presented in Fig. 1. To assess changes across the three timepoints pre-chemotherapy (baseline), at 3 months post-chemotherapy and at 6 months post-chemotherapy, a repeated measures ANOVA was performed. The analysis demonstrated a significant time effect for AMH (F=2.56, P<0.001), E_2_ (F=0.997, P=0.003) and FSH (F=3.227, P<0.001). Post-hoc pairwise comparisons with Bonferroni adjustment revealed a significant decline in AMH levels between baseline and 3 months with a mean difference of 0.938 ng/ml (P<0.001) and between baseline and 6 months with a mean difference of 1.511 ng/ml (P=0.004), as well as between 3 and 6-months post-chemotherapy with a mean difference of 0.573 ng/ml (P=0.025). By contrast, the E_2_ levels demonstrated a significant decrease from baseline to 3 months with a mean difference of 0.222 IU/ml (P 0.013) and from baseline to 6 months with a mean difference of 0.300 IU/ml (P=0.004), and with a further reduction observed between 3 and 6 months, although not statistically significant [mean difference of 0.121 IU/ml (P=0.177] ). FSH levels on the other hand, exhibited a significant increase from baseline to 3 months with a mean difference of -19.522 pg/ml (P<0.001), and from baseline to 6 months with mean difference of -34.202 pg/ml (P=0.004); however, no further significant increase was evident between 3- and 6-months post-chemotherapy with mean difference of -14.680 pg/ml (P=0.076).

The effects of the two chemotherapy combinations on hormone levels were analyzed using ANOVA and paired t-tests. The analysis on the AC/CAF chemotherapeutic regimen using repeated measures ANOVA (pre-chemotherapy vs. 3 months post-chemotherapy, and 3 vs. 6 months post-chemotherapy) yielded the following results: AMH (F=1.688, P=0.064), E_2_ (F=2.111, P=0.073) and FSH (F=1.711, P=0.172). With the chemotherapeutic regimens of taxane + xeloda and platinum compounds + taxane, a paired t-test analysis at two specific intervals of 3 months post-chemotherapy vs. 6 months-post chemotherapy yielded P-values >0.05 (Table VI, Table VII and Table VIII). The mean values were recorded, as well as the mean differences in percentages as presented on the tables. Across all three hormones, there were no significant differences in hormonal changes attributable to the chemotherapy combinations (all P-values >0.05). These data indicated that the type of chemotherapeutic regimen did not significantly influence ovarian hormone response over time.

The effects of chemotherapy on the menstrual cycle of the participants differed slightly according to age. A Fisher's exact test was conducted to assess the association between age group and menstruation patterns at 6 months following chemotherapy. The association was not statistically significant (P=0.146) (Table IX). Some participants experienced changes in their menstrual patterns at 6 months following chemotherapy. At the 12-month follow-up, the menstrual pattern was as follows: Regular, 38 (46%); irregular, 19 (23%); while 25 (31%) exhibited the complete cessation of menstruation.

## Discussion

The diagnosis of breast cancer is increasing and is likely to occur prior to the completion of creating a family in numerous females. The use of chemotherapy is an integral part of the treatment of breast cancer among most pre-menopausal women. However, its administration is not without significant reproductive side-effects in those who are still within their reproductive age group. Understanding the impact of chemotherapy on future fertility is of utmost importance. The present study demonstrated that chemotherapy significantly affected key hormones that regulate the reproductive cycle of pre-menopausal women being treated with chemotherapy for breast cancer. In the present study, three hormones (AMH, E_2_ and FSH) were evaluated for the effects of chemotherapy on the plasma concentrations of these hormones. The mean values of both AMH and E_2_ progressively decreased within the 6 months of their evaluation, while the mean value of FSH increased (Table V). These derangements suggest that the fertility of these pre-menopausal women had been negatively affected. These findings are in agreement with those of previous studies reporting a significant decrease in E_2_ and AMH levels following chemotherapy for breast cancer (17,20). On the other hand, the plasma level of FSH increased following chemotherapy. This is consistent with the findings of previous reports demonstrating reduced ovarian function (21,22). These findings thus support the need for ovarian protection during chemotherapy. There are studies demonstrating that the administration of hormonal therapy (aromatase inhibitor/tamoxifen), particularly in hormone receptor-positive women, or gonadotropin-releasing hormone (GnRH) agonist e.g., goserelin, administered during chemotherapy exerts some degree of protection on the ovaries (23,24).

The present study also indicated changes in the monthly menstrual pattern of some of the participants at 6 months following treatment. Although the quantity of monthly menstrual flow was not measured, the quantity of flow was estimated with fair accuracy, particularly by younger patients, since individuals are already conversant with their menstrual flow pattern (25). In addition, using the records of the number of sanitary pads used during each menstrual period and the duration of flow as recorded by the participants, the authors deem that the quantity of monthly flow was fairly estimated. Almost 15% of the participants became amenorrhoeic following chemotherapy, while 26% developed irregular menstrual patterns (Table IX). This is less than the previously reported proportion of 21 to 71% in young women treated with chemotherapy, although these data are from other populations (26). The data may also reflect the higher number of young patients in the present study. It is also worthwhile to note that those who were ≤30 years of age did not experience the absence of menstruation even though the number was low. A previous study in Nigeria indicated that ~33% of the participants experienced the cessation of the menstrual cycle (27). In that study, participants with various malignancies with different chemotherapeutic regimens were included, whereas the present study only included patients with breast cancer patients with a fairly uniform chemotherapeutic regimens. However, this result is indicative of the higher ovarian reserve in younger patients than older ones following chemotherapy.

In the present study, the effects of the different chemotherapeutic regimens on the expression of the FSH, E_2_ and AMH hormone levels during the study period were also assessed, with no significant differences observed (Table VI, Table VII and Table VIII). The chemotherapeutic regimens used were standard combination regimens used for breast cancer administered every 3 weeks sequentially. All patients received either of the two combinations (anthracyclines + cyclophosphamide) first and then shifted to the second combination (taxanes and platinum compounds) or vice versa. At the end, they were exposed to similar chemotherapeutic agents based on their body surface areas. Thus, the authors deem that the results reflected the cumulative effects of chemotherapy. The analyses did not reveal significant effects of the three regimens on the hormonal levels. This demonstrates that in the present study, the effects of chemotherapy on ovarian function were not dependent on the breast cancer chemotherapeutic regimen administered. This is similar to the previous study by Goldfarb *et al* (28) who reported no difference in the decline of AMH levels among pre-menopausal women receiving cyclophosphamide methotrexate and the 5 fluorouracil (CMF) combination; adriamycin, cyclophosphamide and taxane (AC-T) combination, and then taxane and herceptin (TH) for breast cancer. In addition, Goldfarb *et al* (28) also observed that age was an important predictor of AMH levels as the older age of the participants was associated with lower AMH values. The present study also yielded similar findings.

Almost one third of the participants had hormone-positive breast cancer and were treated with tamoxifen. This may affect the hormonal levels; however, a previous study reported no significant difference in the levels of E_2_ post-chemotherapy and post-hormonal therapy as the patients in each group had decreased E_2_ levels following treatment (17).

A notable number of the participants (77%) preferred fertility preservation; however, they could not afford goserelin, which is the only form of fertility preservation currently available in Nigeria. Some patients had comorbidities, namely hypertension, diabetes and HIV infection, and all were well controlled. A previous study demonstrated that HIV infection does not have significant effect on estrogen levels in pre-menopausal HIV-positive women with an unsuppressed CD4 count (29). The observed changes may mainly be due to the chemotherapy received by the participants. It was also observed that at 6 months post-chemotherapy, none of the hormonal levels indicated recovery from the effects of chemotherapy, indicating that the effects last far beyond 6 months post-treatment. This was supported by the observation that at 12 months, those with regular menses were reduced from 49 (58%) to 38 (46%), while 25 (31%) of the participants experienced cessation of menstrual flow compared with 12 (16%) at 6 months. A longer period of follow-up is required to identify the time and pattern of recovery for those whose ovarian function recovered from the effects of chemotherapy. From the findings of the present study, it is highly recommended that fertility preservation services, such as the use of oocyte/embryo cryopreservation, ovarian suppression and GnRH agonists with counselling be provided to patients scheduled to undergo cancer chemotherapy if they still desire to have children folowing treatment.

### Limitations of the study

The lack of data comparing study completers and non-completers is a limitation to the present study. This could not allow the assessment of the characteristics of those who completed the study against those who did not. Nevertheless, the results obtained still provide useful information about the population under study. However, multivariate analysis adjusting for potential confounders (e.g., age, parity and baseline AMH) was not performed on the study data. A simple analysis was used, since this was within a specific age group of <42 years, and we looked for characteristics of 30 years and >30 years based on previous reports on the literature (30,31). Furthermore, since individual hormonal levels were measured at baseline and then serially, each patient stood as a self-control. Additionally, hormone level variability exists across menstrual phases, and this may have confounded the E_2_ and FSH results. The patients were on 3 weekly chemotherapy treatments, with some participants residing at a distance from the hospital. Measuring the hormone levels based on the menstrual cycle phase would have necessitated more visits to the clinic, which would have been too stressful for the patients with breast cancer receiving chemotherapy. The exact volume of menstrual flow per cycle could not be measured for the purpose of determining reduction in menstrual flow following chemotherapy. However, using the number of sanitary pads used during each menstrual cycle and recording the duration in a diary was deemed to have provided a fair estimate of the quantity of menstrual blood loss by an individual during the study.

The present study was conducted in southwest Nigeria, which represents a fraction of the Nigerian population. The results cannot therefore be generalized to the entire population of Nigeria. Nevertheless, the results provide useful information that can be used to guide the design of larger studies that can give information about the Nigerian population.

In conclusion, 82 Nigerian participants with a pre-menopausal onset of breast cancer treated with chemotherapy were analyzed in the present study. The findings revealed that chemotherapy led to a significant reduction in ovarian function, as reflected by the negative changes in the plasma concentrations of E_2_, FSH and AMH. The effects were more pronounced in participants >30 years of age, as some developed an irregular or cessation of menstrual flow. The different chemotherapeutic regimens for breast cancer did not significantly affect ovarian function. The hormonal levels did not exhibit a recovery at 6 months post-chemotherapy, while the induced reduction in menstrual patterns persisted for 12 months following chemotherapy. These findings suggest that the effects of chemotherapy last >6 months. Clinicians should thus be aware of the deleterious effects of chemotherapy on ovarian function and counsel such patients who desire childbearing appropriately on fertility issues to ensure good survivorship.

## Figures and Tables

**Table I:** Hormonal changes throughout the menstrual cycle.

Phase of menstrual cycle	Estrogen (pg/ml)	Progesterone (ng/ml)	LH (iu/l)	FSH (iu/l)	AMH (ng/ml)
Phase I (menstrual phase- days 1-5)	160.00±66.24	0.60±0.40	7±6.00	1.00-9.00	0.29-12.70
Phase II (follicular phase- days 10-13)	778.00±255.43	0.64±0.31	20.00±8.00	6.00-26.00	2.41 (0.15-14.50)
Phase Ill (luteal phase-days 20-23)	395.00±134.57	14.00±5.44	7.00±4.00	1.00-9.00	2.07 (0.08-8.58)

Data are presented as the mean ± SD. LH, luteinizing hormone; FSH, follicle stimulating hormone; AMH, anti-Müllerian hormone.

**Table II:** Biodata of the study participants.

Variable (n=82)	Frequency	Percentage
Age at presentation (years)		
≤30	10	12.2
31-40	56	68.3
41-50	16	19.5
Mean age, 36.6±4.6		
Occupation		
Skilled professional	9	11.0
Minimally skilled^a^	58	70.7
Not in active employment^b^	15	18.3
Education level		
None	6	7.3
Primary	2	2.4
Secondary	41	50.0
Tertiary	33	40.2
Marital status		
Married	55	67.1
Not married^c^	27	32.9
Partner's occupation (n=55)		
Skilled professional	3	5.5
Minimally skilled^a^	50	90.9
Not in active employment^b^	2	3.6
Parity		
0	24	29.3
1	10	12.2
2	18	22.0
3	13	15.9
≥4	17	20.6
Desire for more children		
Yes	42	51.2
No	40	48.8

^a^Trader, civil servant, artisan;

^b^student, unemployed;

^c^divorced, separated, single, widowed.

**Table III:** Clinical history of the study participants.

Variable (n=82)	Frequency	Percentage
Disease stage		
I	4	4.9
II	7	8.5
III	71	86.6
ECOG score at presentation		
0	47	57.3
1	35	42.7
Nutritional status (BMI) (kg/m^2^)		
Normal	43	52.4
Overweight and obese	39	47.6
Primary site of disease		
Left	46	58.5
Right	36	41.5
Presence of comorbidities		
Yes	12	13.4
No	70	86.6
Type of comorbidities (n=12)		
Hypertension	4	33.3
Peptic ulcer disease	4	33.3
HIV/AIDS	4	33.3
History of breast surgery		
Yes	29	35.4
No	53	64.6
Surgery type (n=29)		
Lumpectomy	9	31.0
Mastectomy	20	69.0

BMI, body mass index; HIV/AIDS, human immunodeficiency virus/acquired immunodeficiency syndrome.

**Table IV:** Chemotherapeutic regimens among the study participants.

Regimen (n=82)	Frequency	Percentage
Phase I		
Anthracycline + cyclophosphamide regimen (AC/CAF)	81	98.8
Taxane + anthracycline + cyclophosphamide (TAC)	1	1.2
Phase II		
Anthracycline + Cyclophosphamide regimen (AC/CAF)	61	74.4
Taxane + xeloda (TX)	13	15.8
Platinum + taxane (PT)	8	9.8

**Table V:** Levels of hormonal parameters among the study participants.

Variable (n=82)	Pre-chemotherapy	Post-chemotherapy (3 months)	Post-chemotherapy (6 months)
AMH (ng/ml)			
<1	46 (56.1)	69 (84.1)	80 (97.6)
1-2	16 (19.5)	6 (7.1)	2 (2.4)
>2	20 (24.4)	7 (8.5)	0 (0.0)
Mean (SD)	1.66 (0.84)	0.71 (0.60)	0.14 (0.16)
E2 (IU/ml)			
<50	57 (69.5)	61 (74.4)	62 (75.6)
50-100	20 (24.4)	18 (22.0)	19 (23.2)
>100	5 (6.1)	5 (6.1)	1 (1.2)
Mean (SD)	57.56 (5.32)	43.15 (4.66)	33.82 (3.37)
FSH (pg/ml)			
<50	61 (74.4)	55 (67.1)	43 (52.4)
50-100	11 (13.4)	14 (17.1)	21 (25.6)
>100	10 (12.2)	13 (15.9)	18 (22.0)
Mean (SD)	14.16 (2.39)	33.70 (4.18)	47.13 (5.29)

LH, luteinizing hormone; FSH, follicle stimulating hormone; AMH, anti-Müllerian hormone.

**Table VI:** Effect of chemotherapy on AMH levels among the study participants.

	1st to 3rd month (mean values)				3rd to 6th month (mean values)			
Drugs	No. of patients	Pre-chemotherapy	Post-chemotherapy (3 months)	Mean difference (%)	No. of patients	Post-chemotherapy (3 months)	Post-chemotherapy (6 months)	Mean difference (%)
AC/CAF	81	1.67 (ng/ml)	0.72 (ng/ml)	0.95 (56.9)	61	0.72 ng/ml	0.16 ng/ml	0.56 (77.8)
						(F=1.688, P=0.064)		
TAC	1	0.82 (ng/ml)	0.11 (ng/ml)	0.71 (86.6)				
		P=0.132						
Taxane + xeloda					13	0.31 (ng/ml)	0.10 (ng/ml)	0.21 (67.7)
						P=0.224		
PT					8	0.52 (ng/ml)	0.09 (ng/ml)	0.43 (82.7)
						P=0.816		

AMH, anti-Müllerian hormone; AC/CAF, anthracycline + cyclophosphamide; TAC, taxane + anthracycline + cyclophosphamide; PT, platinum + taxane.

**Table VII:** Effect of chemotherapy on E_2_ levels among the study participants.

	1st to 3rd month (mean values)				3rd to 6th month (mean values)			
Drugs	No. of patients	Pre-chemotherapy	Post-chemotherapy (3 months)	Mean difference (%)	No. of patients	Post-chemotherapy (3 months)	Post-chemotherapy (6 months)	Mean difference (%)
AC/CAF	81	53.08 (IU/ml)	38.70 (IU/ml)	14.38 (27.1)	61	38.70 (IU/ml)	36.15 (IU/ml)	2.55 (6.58)
						(F=2.111, P=0.073)		
TAC	1	57.56 (IU/ml)	43.15 (IU/ml)	14.41 (25.0)		43.15 (IU/ml)	33.82 (IU/ml)	9.35 (21.7)
		P=0.418				P=0.079		
Taxane + xeloda					13	28.81 (IU/ml)	25.09 (IU/ml)	3.72 (12.9)
						P=0.827		
PT					8	32.90 (IU/ml)	21.09 (IU/ml)	11.81 (35.9)
						P=0.308		

E_2_, estradiol; AC/CAF, anthracycline + cyclophosphamide; TAC, taxane + anthracycline + cyclophosphamide; PT, platinum + taxane.

**Table VIII:** Effect of chemotherapy on FSH levels among the study participants.

	1st to 3rd month (mean values)				3rd to 6th month (mean values)			
Drugs	No. of patients	Pre-chemotherapy	Post-chemotherapy (3 months)	Mean difference (%)	No. of patients	Post-chemotherapy (3 months)	Post-chemotherapy (6 months)	Mean difference (%)
AC/CAF	81	14.22 (pg/ml)	34.03 (pg/ml)	19.81 (139.3)	61	34.03 (pg/ml)	42.77 (pg/ml)	8.74 (25.68)
						(F=1.711, P=0.172)		
TAC	1	8.87 (pg/ml)	7.25 (pg/ml)	-1.62 (-18.3)				
		P=0.938						
Taxane + xeloda					13	41.46 (pg/ml)	49.51 (pg/ml)	8.05 (19.4)
						P=0.177		
PT					8	46.12 (pg/ml)	83.21 (pg/ml)	37.09 (80.4)
						P=0.853		

FSH, follicle-stimulating hormone; AC/CAF, anthracycline + cyclophosphamide; TAC, taxane + anthracycline + cyclophosphamide; PT, platinum + taxane.

**Table IX:** Association between age and menstruation patterns (at 6 months following chemotherapy).

	Menstruation pattern				
	Regular (%)	Irregular (%)	Absent (%)	Total	P-value
Age (years)					0.146
≤30	6(7)	4(5)	0 (0.0)	10	
31-40	37(45)	11(14)	8(10)	56	
41-50	6(7)	6(7)	4(5)	16	

## Data Availability

The data generated in the present study may be requested from the corresponding author.
